# Supplementary material for: Economic evaluation of diagnostic tests for Thai patients with tuberculosis: A dynamic transmission model approach
Source: PLoS One. 2025 Mar 3;20(3):e0315772. doi: 10.1371/journal.pone.0315772 (PMC11875360; doi:10.1371/journal.pone.0315772)
Supplement: S1 Table — (PDF) [file pone.0315772.s001.pdf]

Table S1 Model Parameters.

Parameter detail	Mean	Standard Error	References
**Diagnostic performance**			
Sensitivity of SSM	0.6100	0.1480	[1]
Specificity of SSM	0.9800	0.0179	[1]
Sensitivity of liquid culture for smear-negative TB	1.0000	-	Assumption
Specificity of liquid culture for smear-negative TB	1.0000	-	Assumption
sensitivity of DST	1.0000	-	Assumption
specificity of DST	1.0000	-	Assumption
Sensitivity of Xpert MTB/RIF followed smear negative	0.6800	0.0332	[2]
Specificity of Xpert MTB/RIF followed smear negative	0.9900	0.0026	[2]
Sensitivity of Xpert MTB/RIF Initial testing	0.8800	0.0204	[2]
Sensitivity of Xpert MTB/RIF Initial testing	0.9900	0.0026	[2]
Sensitivity of Xpert MTB/RIF for detection RR	0.9500	0.0179	[2]
Sensitivity of Xpert MTB/RIF for detection RR	0.9800	0.0051	[2]
Sensitivity of TB-LAMP smear negative	0.4030	0.0666	[3]
Specificity of TB-LAMP smear negative	0.9770	0.0064	[3]
Sensitivity of TB-LAMP all smear	0.8030	0.0439	[3]
Specificity of TB-LAMP all smear	0.9770	0.0066	[3]
**Mortality rate of active TB**			
Death rate of untreated TB patient	0.50	0.0469	Model calibration
Death rate of pan-sensitive TB-patient	0.056	0.0026	Model calibration
Death rate of MDR-TB patient	0.110	0.0110	[4]
Death rate of XDR-TB patient	0.250	0.0250	[4]
Risk of TB activation	0.031	0.0046	[5]

Parameter detail	Mean	Standard Error	References
Rate of active TB among LTBI per month	0.054	0.0015	Model calibration
Rate of conversion from smear negative to smear positive TB per month	0.50	0.1148	Model calibration
rate of self-care without any treatment per month	0.077	0.0005	Model calibration
Probability of treatment success in new and relapse TB with first line drug regimen	0.8520	0.0006	[4]
Probability of treatment success in new and relapse MDR-TB with MDR/RR-drug regimen	0.5746	0.0087	[4]
Probability of treatment success in new and relapse XDR-TB with XDR-drug regimen	0.6250	0.0823	[4]
Rate of acquisition of TB drug resistance from pan-sensitive to MDR-TB	0.0019	0.0004	[4]
Rate of acquisition of TB drug resistance from MDR TB to XDR-TB	0.0192	0.0039	[6]
Case detection rate per month	0.90	-	Expert opinion
Proportion of pan-sensitive TB in all active TB	0.9890	0.0003	[4]
Proportion of MDR-TB in all active TB	0.0107	0.0002	[4]
Proportion of XDR-TB in all active TB	0.0003	0.0001	[4]
Lost to follow up after diagnosis by DST	0.10	-	Expert opinion
**Costs in 2022 (Baht)**			
Unit cost of CXR	200	20	[7]
Unit cost of SSM	70	25	
Unit cost of Culture	200	7	
Unit cost of DST	250	20	
Unit cost of Xpert MTB/RIF	880	88	
Unit cost of TB-LAMP	880	88	
Unit cost of LPA	1500	20	

Parameter detail	Mean	Standard Error	References
Cost of OPD service	259	26	
First line drug treatment per month<br>- intensive phase: HRZE<br>- continuous phase: HR	 482<br>189	 48<br>19	[8]
MDR regimen (shorter regimen) per month<br>- intensive phase (4 months): Km Mfx Pto Cfz Z E H<br>- continuous phase (5 months): Mfx Cfz Z E	 4,259<br>3,533	 426<br>353	
XDR regimen (shorter regimen) per month: 8 Cm 12Lzd 20Cfz 20Mfx 6Bdq<br>- intensive phase (8 months)<br>- continuous phase (12 months)	 61,488<br>15,210	 6,149<br>1,521	
Travel cost, accommodation cost and food	527.78	73.12	[9]
Formal care cost	6.32	3.47	
Travel cost, accommodation cost and food<br>- DS-TB<br>- MDR-TB	 2,877.52<br>543.46	 250.68 143.58	
Formal care cost<br>- DS-TB<br>- MDR-TB	 64.81<br>3.73	 25.42<br>3.74	
**Health Utilities**			
Ongoing DS-TB treatment	0.6900	0.04	[10]
Ongoing MDR-TB treatment	0.5100	0.11	
Ongoing XDR-TB treatment	0.5100	0.11	
Treated/complete TB treatment	0.8800	0.06	

CXR indicates chest X-ray; DS-TB, drug sensitive tuberculosis; MDR-TB, multidrug-resistant tuberculosis; XDR-TB, extensive-resistance tuberculosis; RR, rifampicin-resistant tuberculosis; DST, drug susceptibility testing; FL-DST, first line- drug susceptibility testing; SL-DST, second line- drug susceptibility testing; SSM, sputum smear microscopy; Bdq, bedaquiline; Cfz, clofazimine; Cm, capreomycin; E, ethambutol; H indicates isoniazid; Km, kanamycin; Lfx, levofloxacin; Lzd, lizenolid; Mfx, moxifloxacin; Pto, prothionamide; R, rifampicin; Z, pyrazinamide.

References

1. World Health Organization. Systematic screeening for active tuberculosis: an operation guide. Geneva: World Health Organization; 2015.

2. World Health Organization. Policy update: Xpert MTB/RIF assay for the diagnosis of pulmonary and extrapulmonary TB in adults and children. Geneva: World Health Organization; 2017.

3. Shete PB, Farr K, Strnad L, Gray CM, Cattamanchi A. Diagnostic accuracy of TB-LAMP for pulmonary tuberculosis: a systematic review and meta-analysis. BMC Infect Dis. 2019;19(1):268.

4. Tuberculosis country profiles (Thailand) [Internet]. The World Health Organization. 2023 [cited March 20, 2024]. Available from: https://www.who.int/tb/country/data/profiles/en/.

5. Fox GJ, Barry SE, Britton WJ, Marks GB. Contact investigation for tuberculosis: a systematic review and meta-analysis. Eur Respir J. 2013;41(1):140-56.

6. Menzies NA, Cohen T, Lin H-H, Murray M, Salomon JA. Population health impact and cost-effectiveness of tuberculosis diagnosis with Xpert MTB/RIF: a dynamic simulation and economic evaluation. PLoS Med. 2012;9(11):e1001347-e.

7. Cost of Health Services: The Comptroller General's Department, Thailand.; [cited 2022. Available from: https://mbdb.cgd.go.th/wel/searchmed.jsp.

8. the median drug price 2019 [Internet]. Drug And Medical Supply Information Center, Ministry of Public Health. 2019. Available from: http://dmsic.moph.go.th/dmsic/index.php?p=1&type=3&s=3&id=middle_drug.

9. Youngkong S, Thavorncharoensap M, Chaikledkaew U, Jittikoon J, Praditsitthikorn N, Mahasirimongkol S, et al. Economic burden of out-of-pocket payment for healthcare

expenditures of tuberculosis patients in Thailand (2st Year). Health System Research Institue, Thailand: Health System Research Institue, Thailand, Health System Research Institue T; 2022.

10. Kittikraisak W, Kingkaew P, Teerawattananon Y, Yothasamut J, Natesuwan S, Manosuthi W, et al. Health related quality of life among patients with tuberculosis and HIV in Thailand. PLoS One. 2012;7(1):e29775-e.

11. Posuwan N, Vuthitanachot V, Chinchai T, Wasitthankasem R, Wanlapakorn N, Poovorawan Y. Serological evidence of hepatitis A, B, and C virus infection in older adults in Khon Kaen, Thailand and the estimated rates of chronic hepatitis B and C virus infection in Thais, 2017. PeerJ. 2019;7:e7492.
