# Supplementary material for: Economic evaluation of diagnostic tests for Thai patients with tuberculosis: A dynamic transmission model approach
Source: PLoS One. 2025 Mar 3;20(3):e0315772. doi: 10.1371/journal.pone.0315772 (PMC11875360; doi:10.1371/journal.pone.0315772)
Supplement: Attachment — Submitted filename: Response to Reviewers-29Nov2024.docx [file pone.0315772.s002.docx]

**Responses to Editors and Reviewers**

**Journal Requirements:**

1. When submitting your revision, we need you to address these additional requirements.

Please ensure that your manuscript meets PLOS ONE's style requirements, including those for file naming. The PLOS ONE style templates can be found at 
https://journals.plos.org/plosone/s/file?id=wjVg/PLOSOne_formatting_sample_main_body.pdf and https://journals.plos.org/plosone/s/file?id=ba62/PLOSOne_formatting_sample_title_authors_affiliations.pdf

**Response:** Thank you for your suggestion. We ensure that our manuscript meets PLOS ONE’s style requirements, including those for file naming.

2. Please note that PLOS ONE has specific guidelines on code sharing for submissions in which author-generated code underpins the findings in the manuscript. In these cases, we expect all author-generated code to be made available without restrictions upon publication of the work. Please review our guidelines at https://journals.plos.org/plosone/s/materials-and-software-sharing#loc-sharing-code and ensure that your code is shared in a way that follows best practice and facilitates reproducibility and reuse.

**Response:** Thank you for your suggestion. All data have been reported in our manuscript and supporting information.

3. Thank you for stating the following financial disclosure:

“We would like to acknowledge the Thailand Science Research and Innovation under the Ministry of Higher Education, Science, Research and Innovation for providing the Royal Golden Jubilee Ph.D. scholarship for NC and UC (grant no. PHD/0104/2559). This research project has been funded by the Health System Research Institute (HSRI), Thailand as well as Mahidol University (Fundamental Fund: fiscal year 2024) by National Science Research and Innovation Fund (NSRF). The findings, interpretations and conclusions expressed in this article do not necessarily reflect the views of the aforementioned funding agencies.”

Please state what role the funders took in the study. If the funders had no role, please state: "The funders had no role in study design, data collection and analysis, decision to publish, or preparation of the manuscript."

If this statement is not correct you must amend it as needed.

Please include this amended Role of Funder statement in your cover letter; we will change the online submission form on your behalf.

**Response:** Thank you for your kind suggestion. We have included this amended Role of Funder statement in our cover letter.

4. Your ethics statement should only appear in the Methods section of your manuscript. If your ethics statement is written in any section besides the Methods, please move it to the Methods section and delete it from any other section. Please ensure that your ethics statement is included in your manuscript, as the ethics statement entered into the online submission form will not be published alongside your manuscript.

**Response:** Thank you for your helpful suggestions. The ethics statement has been moved to the Methods section (Materials and methods, line 110-117, page 5-6).

5. Please review your reference list to ensure that it is complete and correct. If you have cited papers that have been retracted, please include the rationale for doing so in the manuscript text, or remove these references and replace them with relevant current references. Any changes to the reference list should be mentioned in the rebuttal letter that accompanies your revised manuscript. If you need to cite a retracted article, indicate the article’s retracted status in the References list and also include a citation and full reference for the retraction notice.

**Response:** Thank you for your helpful suggestions. We have reviewed our reference list and ensured that it is complete and correct. We have already removed the incorrect reference and added the correct reference.

**Reviewers' comments**

**Reviewer #1**

The manuscript by Chitpim et al. provides a well-structured analysis of the cost-utility of TB diagnostics in Thailand and has used appropriate methodology. Overall, the results support the main conclusion that molecular tests, such as Xpert, are more cost-effective than conventional methods. The findings offer valuable evidence for policymakers, particularly in advocating for the integration of Xpert MTB/RIF into national health programs.

**Response:** We would like to thank a reviewer for your valuable comments and suggestions. We feel that the revised paper is much further improved as a consequence of your inputs.

**Additional Comments:**

• In line 69, reference number 8 appears to be cited incorrectly.

**Response:** Thank you very much for pointing this out. We apologize for a citing error. We have already deleted reference number 8 and cited reference number 1 as follows (Line 68, page 3).

1. National Tuberculosis Program guideline, Thailand: Bureau of Tuberculosis, Ministry of Public Health; 2018.

• Line 132 has a typo: "Tjpse" should be "those."

**Response:** Thank you very much for your very helpful suggestion. We apologize for a typing error. We have corrected as suggested (Line 139, page 7).

• Line 369 has a typo: “which have used static models” should be “which has used.”

**Response:** Thank you very much for your very helpful suggestion. We apologize for a typing error. We have corrected as suggested (Line 374, page 20).

• While the limitations are well-stated, consider suggesting potential solutions or directions for future research to address these limitations.

**Response:** Thank you very much for your very helpful suggestion. We have revised the paragraph addressing the limitations and directions for future research as follows (Discussion, line 376-386, page 20).

*“Firstly, it did not include the costs associated with TB re-diagnosis across all health states, potentially underestimating the total economic burden, especially in cases of treatment failure or relapse. Future studies should incorporate these costs for a more comprehensive evaluation. Secondly, the assumption of 100% medication adherence does not align with real-world scenarios. Future models reflecting varied adherence rates would provide more realistic insights into treatment outcomes and associated costs. Thirdly, the study presumed uniform natural recovery rates after smear microscopy, which may oversimplify the diversity of patient outcomes. Future research should explore a range of recovery rates to capture real-world variability. Lastly, there was a lack of data on utility parameters before and at the time of diagnosis. Incorporating these parameters in future studies would yield a more understanding of TB’s impact on quality of life.”*

**Reviewer #2**

Grammatical and Typographical Errors

**Response:** We would like to thank a reviewer for your valuable comments and suggestions. We feel that the revised paper is much further improved as a consequence of your inputs.

- in Line 132: “Tjpse” should be corrected to “Those.”

**Response:** Thank you very much for your very helpful suggestion. We apologize for a typing error. We have corrected as suggested (Line 139, page 7).

- Word Choice in Line 297: “Incremental Costs” might be more consistently phrased as "Incremental Cost" in Table 1, as it’s commonly singular in economic analyses.

**Response:** Thank you very much for your great suggestion. We have changed from “Incremental Costs” to “Incremental Cost” (Table 1, line 300, page 16).

-Sentence fragment in Line 296: The phrase “Dominant indicates lower costs and higher QALYs” could be more readable if rephrased to clarify that dominance in this context refers to both lower costs and improved effectiveness or increased QALYs.

**Response:** Thank you very much for your great suggestion. We have rephrased to “Dominance in this context refers to both lower costs and improved effectiveness or increased QALYs.” (Table 1, line 301, page 16).

While revising your submission, please upload your figure files to the Preflight Analysis and Conversion Engine (PACE) digital diagnostic tool, https://pacev2.apexcovantage.com/. PACE helps ensure that figures meet PLOS requirements. To use PACE, you must first register as a user. Registration is free. Then, login and navigate to the UPLOAD tab, where you will find detailed instructions on how to use the tool. If you encounter any issues or have any questions when using PACE, please email PLOS at figures@plos.org. Please note that Supporting Information files do not need this step.

Response: Thank you for your helpful suggestions. We have already revised figure files and uploaded figures files PACE as suggested.
